# Retraction Note: LncRNA miR503HG interacts with miR-31-5p through multiple ways to regulate cancer cell invasion and migration in ovarian cancer

**DOI:** 10.1186/s13048-020-00747-z

**Published:** 2021-02-01

**Authors:** Ding Zhu, Xueshuang Huang, Fang Liang, Lijing Zhao

**Affiliations:** 1 Department of Gynecology, Hunan Provincial People’s Hospital, Changsha City, Hunan Province 410005 People’s Republic of China; 2 Biomedical Research Center, Hunan University of Medicine, No.492 Jinxi South Road, Huaihua City, Hunan Province 418000 People’s Republic of China

**Retraction Note: J Ovarian Res 13, 3 (2020)**

10.1186/s13048-019-0599-9

The Editor-in-Chief has retracted this article [1] due to lack of evidence that the study has received ethics approval. After publication it has come to the Editor’s attention that in the body of the article the authors state that their study was approved by the Ethics Committee of Hunan University of Medicine. However, in the Ethics declarations section, the authors state that their study was approved by the Ethics Committee of the Maternity and Child Care Center of Liuzhou. The authors have not provided documentation of approval from an ethics committee for this study. The authors agree with this retraction.
